# The sixth sense of the brain: interoception in organoid study

**DOI:** 10.1038/s41413-026-00569-7

**Published:** 2026-08-17

**Authors:** Jilong Li, Fuxiao Wang, Qianmin Gao, Xiao Chen, Long Bai, Jiacan Su

**Affiliations:** 1 Institute of Translational Medicine, Shanghai University, Shanghai, China; 2 MedEng-X Institutes, Shanghai University, Shanghai, China; 3 National Center for Translational Medicine (Shanghai) SHU Branch, Shanghai University, Shanghai, China; 4 Department of Orthopedics, Xinhua Hospital Affiliated to Shanghai Jiao Tong University School of Medicine, Shanghai, China; 5 Wenzhou Institute of Shanghai University, Wenzhou, China

**Keywords:** Bone quality and biomechanics, Bone

## Abstract

Interoception is the process by which the nervous system senses, integrates, and interprets signals arising from within the body, thereby enabling the brain to monitor and regulate physiological functions. Often described as a “sixth sense”, interoception links internal physiological states to brain-mediated homeostatic control, whereas sight, smell, hearing, taste, and touch primarily convey information from the external environment. Organoids are three-dimensional tissue models derived from stem or progenitor cells that reproduce selected structural and functional features of native organs. Because organoids are increasingly used in disease modeling, drug testing, and regenerative research, their integration with neural, endocrine, immune, and vascular components offers an opportunity to investigate brain-body communication. Conversely, organoid-based systems may provide experimentally tractable models for interoception. This review defines interoception and organoid technologies, examines brain-centered communication with skeletal and intestinal systems, and discusses how brain, bone, and intestinal organoids and assembloids could advance interoception research. In particular, organoid-based models may enable mechanistic investigation of neuroendocrine pathways, including the hypothalamic-pituitary-adrenal (HPA) axis, and thereby provide new insights into physiological regulation and disease progression.

## Introduction

The nervous system processes sensory signals into internal, proprioceptive, and external sensations.^1^ Interoception is a neural process responsible for perceiving, integrating, and transmitting internal physiological signals between the body and the brain.^2,3^ Multiple organs have interoceptive mechanisms and communicate bidirectionally with the brain.^4^ Interoception plays a key role in maintaining functional homeostasis in the brain, bones, and intestines.^4–6^ Interoceptive pathways are usually associated with the central nervous system or endocrine regulation, such as the hypothalamic-pituitary-adrenal (HPA) axis pathway,^7^ which provides potential entry points for interoception research.^2^ The current research on brain, bone, and gut interoception mainly relies on in vitro cell cultures or in vivo models. However, traditional methods have many limitations, including the limited extracellular-matrix context of conventional cell cultures and the cost, throughput, and species-related constraints of in vivo models.

These limitations motivate the development of complementary model systems for studying interoception across organs. Current cellular research mainly focuses on in vitro cultures, in which three-dimensional (3D) culture systems demonstrate significant advantages over conventional 2D cultures. These advantages include: 1) better replication of in vivo microenvironments through extracellular matrix (ECM) modeling; 2) more realistic cell-cell interactions by improving cell junctional communication; 3) enhanced ability to model complex tissues and organs. Since organoid-based three-dimensional cell and tissue culture can simulate the developmental process of tissues in a three-dimensional environment and replicate certain endocrine functions of organs,^8^ organoids have become a powerful platform for studying mechanisms of inter-organ crosstalk.

Organoids are three-dimensional (3D) cell culture models derived from induced pluripotent stem cells or primary tissues and have transformed biological research by mimicking the structure and function of human organs.^9^ Unlike conventional in vitro cultures, they exhibit structural and functional similarities to their target organs. This allows them to replicate physiological functions or model pathological conditions of specific organs.^10^ Organoids play a key role in drug discovery, precision medicine, and regenerative medicine.

**Fig. 1:**
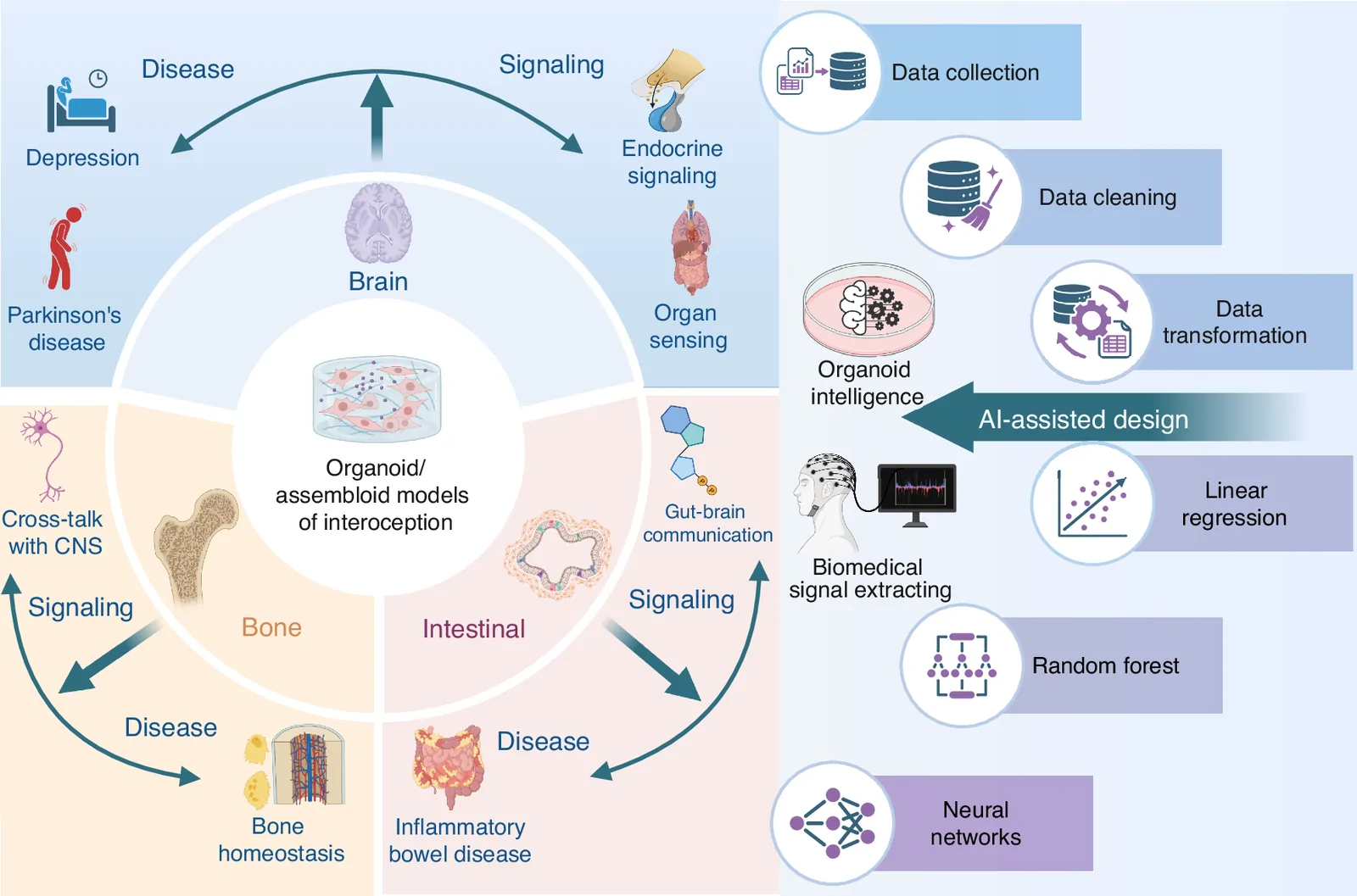
Conceptual intersections between organoid models and interoception research. Brain, bone, and intestinal organoids or assembloids may be integrated with neural and endocrine signaling pathways to model brain-body communication and relevant disorders. The right panel illustrates a prospective AI-enabled workflow for multimodal data collection, preprocessing, modeling, and feedback-guided experimental design

This review focuses on elucidating the potential relationship between interoception and organoids. Firstly, we highlight three types of organoids that demonstrate interoceptive connections with the central nervous system: brain organoids, skeletal organoids, and gut organoids. Secondly, we emphasize their potential relevance to the gut-brain axis, bone-brain axis, and neurological disorders. Additionally, the review delves into the integration of artificial intelligence (AI) with organoid research, exploring novel technological applications. Finally, we discuss current limitations of organoid models and future research directions (Fig. 1).

## Organoids and interoception

### Definition and function of organoids

Organoid research emerged from advances in regenerative medicine, which involves cultivating stem cells to differentiate into multiple mature cell types required for repair, enabling in vitro reconstruction of organs for studying tissues and organs. As early as the 1970s, Howard Green attempted to co-culture 3T3 fibroblasts to form a stratified squamous epithelial cell colonies resembling human epidermis. With a deeper understanding of the extracellular matrix (ECM), subsequent studies employed extracellular-matrix scaffolds that support cell growth and reproduce key matrix cues,^11^ including natural ECM components and synthetic elements.^12,13^ These approaches address the absence of a suitable extracellular matrix, a key determinant of cell behavior, in conventional planar adherent culture. In addition, due to the three-dimensional culture characteristics of organoids, they provided suitable spatial structures that are more conducive to initial cell differentiation and rearrangement into different spatial regions. The final characteristics of organoids depend on the cell source, such as ASCs, PSCs, or fetal progenitor cells.^12^

Organoids are considered “minimal organ models” and can be used in vitro.^12^ In the field of biotechnology, organoids can be used for organ-level disease modeling to screen specific drugs in various disease models. In addition, they can also be used to design organoid tumor biobanks targeting different pathological features. For example, the colorectal cancer organoid biobank can preserve the histopathological subtypes and major genetic features of the primary tumor, demonstrating the potential of cancer organoids.

Another prominent application of organoids lies in regenerative medicine. Organoids have demonstrated the ability to restore organ functionality. For example, transplanted functional colonic epithelial organoids covered damaged mucosa and, after four weeks, generated terminally differentiated epithelial cell types in a mouse model of induced acute colitis. Moreover, studying the spatiotemporal dynamics of organoid transplantation in the mouse colon provides valuable insights for advancing cell therapies.

### Definition and function of interoception

According to mainstream definitions, interoceptive processing can be considered across three components: 1) Sensory cells located in visceral organ walls, mucosal layers, and specialized sensory cells in the peripheral vascular system; 2) Neural transmission pathways including sympathetic and parasympathetic nervous system pathways originating from the spinal cord and thalamus; and 3) Brain regions involving prediction and somatic representation, including the brainstem, subcortical structures, limbic system, paralimbic regions, and cortical areas.^6^

Unlike external sensory perception, such as vision, interoception primarily detects the internal physiological state of the human body, including heart rate, respiratory rate, blood glucose concentration, urination, sweating, etc. It can reflect the internal state of the body, whether in a conscious or unconscious state, and is considered an important component of cognition and emotion, covering impulses, reflexes, feelings, driving forces, and adaptive behaviors. In addition, interoceptive processing integrates predicted states with prior information through Bayesian inference to support closed-loop homeostatic control.^14^ These signals help the brain regulate homeostasis, emotional states, and autonomic nervous responses.^1^

In addition to triggering emotional experiences, a large body of evidence suggests that interoceptive dysfunction is associated with psychiatric and neurodevelopmental disorders such as autism spectrum disorder, anxiety, and depression. Dysregulation of these interoceptive mechanisms may not only lead to organ dysfunction, such as irritable bowel syndrome, but also contribute to emotional disorders, including anxiety and depression. Beyond irritable bowel syndrome and anxiety/depression, interoceptive dysfunction is implicated in additional disorders, including eating disorders (e.g., anorexia nervosa via altered hunger/satiety signals), obesity (impaired energy homeostasis), chronic pain syndromes, and functional neurological disorders (FND). These highlight interoception’s central role in multisystem homeostasis and underscore the need for advanced models like organoids to dissect disease mechanisms.^15^ Exploring the impact of interoception on psychopathology and developing therapies based on interoception will become an important direction for future research.^1,6,16^

## Potential relevance between organoids and interoception

**Fig. 2:**
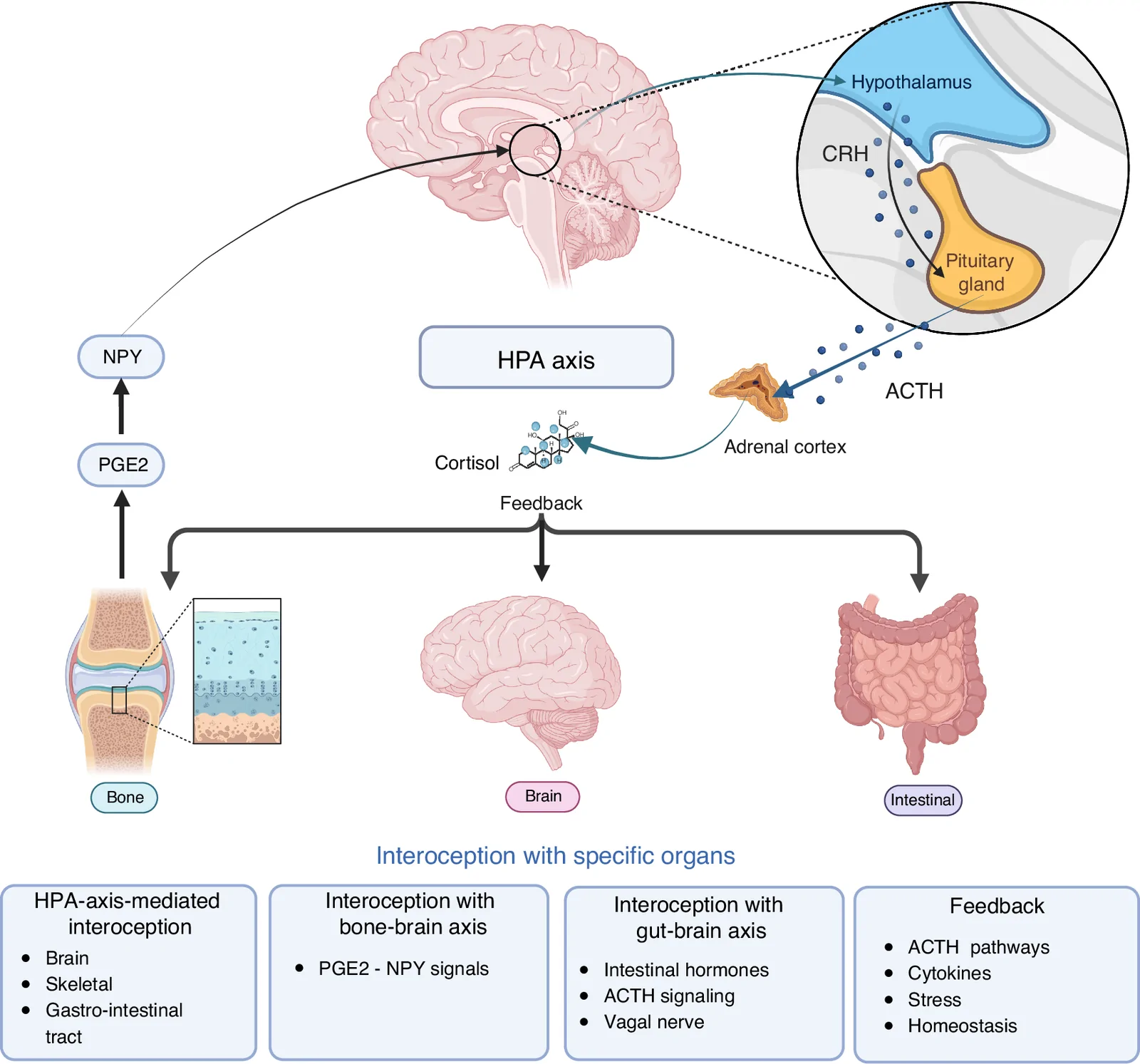
Neuroendocrine and skeletal signals potentially relevant to organoid-based interoception models. The hypothalamic-pituitary-adrenal (HPA) axis links hypothalamic corticotropin-releasing hormone (CRH), pituitary adrenocorticotropic hormone (ACTH), adrenal cortisol secretion, and feedback regulation. Skeletal signaling through the PGE2-EP4-NPY axis is presented as a candidate pathway for modeling bone-brain communication; its integration with organoid or assembloid systems remains an emerging research direction

Within interoceptive circuits, the descending pathway usually includes both neural and non-neural pathways. The former involves spinal sympathetic nerve fibers and parasympathetic vagus nerve fibers, mainly responsible for regulating heart, lung, and gastrointestinal functions. Non-neural signaling also contributes to peripheral regulation through humoral, vascular, and lymphatic routes. In addition, interoceptive processing is influenced by neuroendocrine pathways, including the HPA axis (Fig. 2). These circuits integrate central processing with peripheral sensory receptors, such as chemoreceptors, osmoreceptors, mechanoreceptors, taste receptors, and temperature receptors.^14^ When a person’s behavior and emotional response change, the corresponding interoception signals also change, allowing the body to make timely feedback adjustments until a new dynamic balance is reached.

**Fig. 3:**
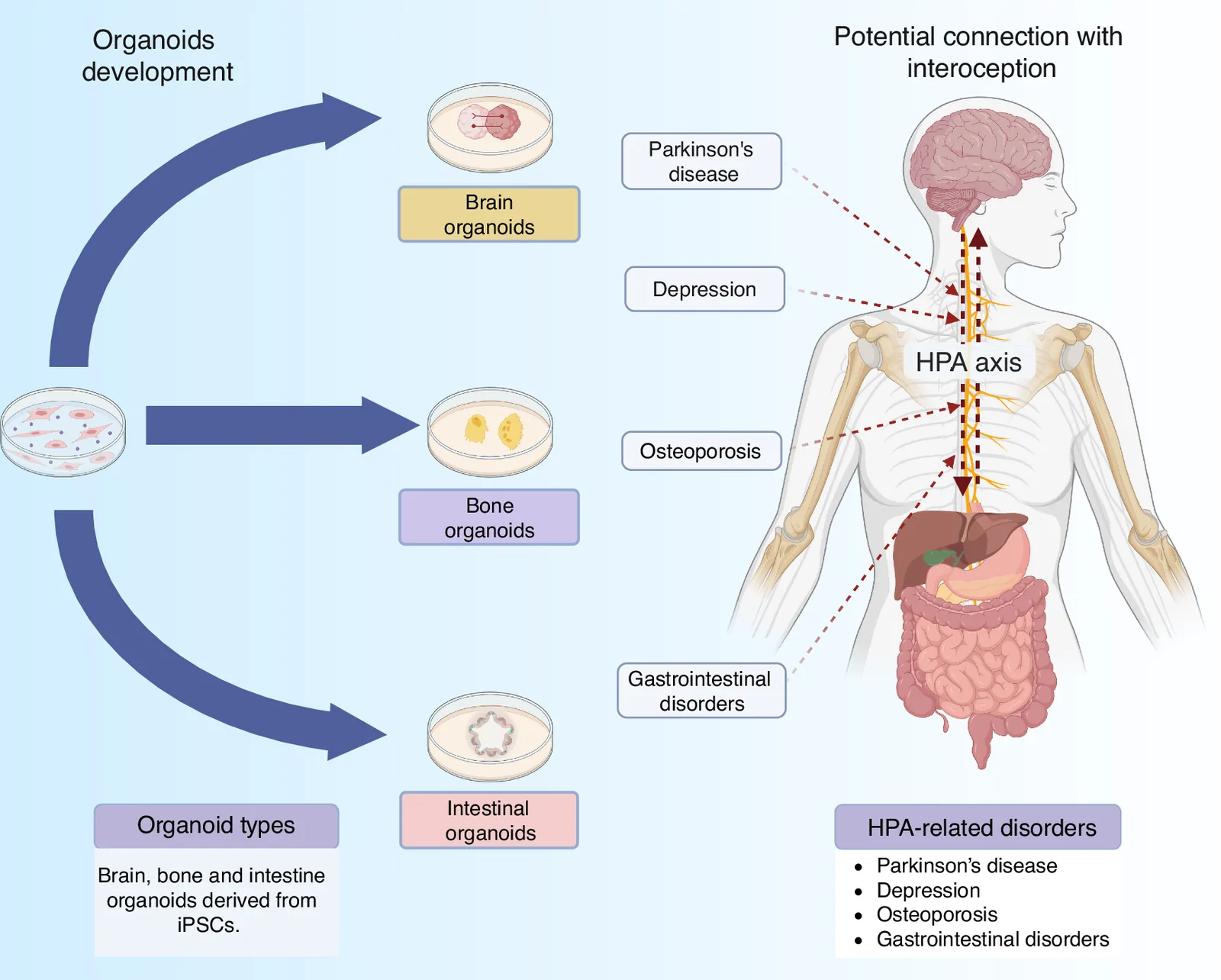
Potential applications of organoids for studying interoceptive regulation. Brain, bone, and intestinal organoids derived from iPSCs may provide complementary models for investigating neuroendocrine and brain-body communication pathways relevant to neurological, skeletal, and gastrointestinal disorders. The HPA axis is shown as one candidate regulatory pathway rather than a unifying causal mechanism for all listed diseases

To investigate factors that may affect interoception, the HPA axis represents one candidate entry point. Organs that interact with the HPA axis include the brain, skeletal system, and gastrointestinal tract.^17–19^ As a new type of three-dimensional cell culture platform, organoids have shown great research and development potential. Brain, skeletal, and gastrointestinal organoids offer complementary experimental opportunities (Fig. 3)

Emerging evidence supports skeletal interoception in vivo; however, modeling PGE2-EP4 signaling to hypothalamic neurons using bone-brain assembloids remains a prospective opportunity.^20^ Similarly, gut organoids integrated with neural components could provide a platform for testing how microbial metabolites influence vagal or enteric signaling.^21^ These models overcome limitations of 2D cultures by preserving 3D architecture and dynamic signaling, supporting the development of experimentally tractable models for organoid-based interoception research.

### Brain

One of the main functions of the brain is to regulate key biological processes such as blood pressure, digestion, and respiration, thereby allowing the body to adapt to changes in both internal and external environments. This regulation relies on the ability to perceive the internal state of the body. Interoception provides the necessary dynamic interaction between the body and the brain, maintaining homeostasis through automatic reflexes such as baroreflexes, motivational drives, and conscious bodily sensations such as shortness of breath, bladder filling, or stomach pain.

The brain receives sensory stimuli from neural routes such as the vagus nerve, spinal cord, and thalamus; the central nervous system transmits inner body signals from internal organs (heart, bones, and digestive tract) to the brain for processing and organizes internal homeostasis through interoceptive networks. Brain regions involved in interoceptive processing include the hypothalamus, nucleus of the solitary tract, thalamus, insular cortex, and prefrontal cortex.

#### Brain organoids and interoception

Psychological studies have shown that interoceptive dysfunction is associated with conditions such as depression, anxiety, and autism spectrum disorder (ASD). Neuroimaging research reveals the crucial role of interoception in regulating human emotional processing and consciousness. A large amount of research has focused on the functional connections between the insular cortex and other primary sensory areas. For example, Ebisch et al.^22^ found that in adult patients with autism spectrum disorder, there is a weakened response or interrupted connection between the anterior and posterior insular regions and the corresponding somatosensory limbic pathway.^4^

Although there is a correlation between neuropathology and interoceptive abnormalities, effective therapeutic interventions are still scarce. This limitation can be partially attributed to the inherent flaws of current pathological models, particularly two-dimensional in vitro cultures and rodent models, which exhibit poor translational efficacy between human and rodent models. Brain organoids have emerged as a promising next-generation platform for establishing precise in vitro pathological models. By integrating specific patterning factors such as bone morphogenetic proteins (BMPs) and Wnt signaling molecules, these organoids exhibit enhanced cellular diversity and compatibility with three-dimensional culture systems. Such protocols can generate region-specific neural tissues from pluripotent stem cells (PSCs) or neural progenitors.^23^

Consequently, organoids facilitate functional simulation, cellular heterogeneity, and precise neuronal connectivity. For example, Kasai et al.^24^ generated functional hypothalamic-pituitary units from 3D-cultured hiPSCs, resembling the hypothalamic-pituitary axis, and demonstrated the ability to respond to corticotropin-releasing hormone (CRH) by secreting adrenocorticotropic hormone (ACTH). This illustrates the potential of brain organoids for studying interoception through the HPA axis. Region-specific organoids (e.g., pituitary organoids and cortical spheroids) have been developed to model distinct brain areas, while more sophisticated whole-brain organoids aim to recapitulate large-scale brain regions. Nevertheless, current brain organoid technology faces notable limitations,^25^ primarily the restricted developmental potential equivalent to prenatal brain maturation stages, coupled with insufficient inter-regional cellular connectivity and diversity.

### Bone

Traditional views held that bones served solely for support and protection, but modern research has revealed that bone also functions as an endocrine organ, with its metabolism regulated by the endocrine system.^26,27^ Hypothalamic endocrine activity plays a critical role in skeletal sensory functions, involving the parathyroid glands, kidneys,^28^ and intestines. Additionally, it promotes phosphate homeostasis during the mineralization of the extracellular matrix of bone. This homeostatic regulation implies crosstalk between the skeleton and these organs.^29^

Bone also plays significant roles in regulating energy metabolism,^30^ immune responses, and neurological functions. By secreting bone-derived factors, such as osteocalcin and bone morphogenetic proteins (BMPs), bone can release these substances into the bloodstream, thereby influencing the functions of distant organs.^31^ For instance, bone-derived osteocalcin crosses the blood-brain barrier, binds GPR158/GPR37 receptors, enhances hippocampal neurogenesis, improves cognition and memory, and reduces anxiety via monoamine modulation.^32^

Evidence supports interoceptive communication between bone and the central nervous system. Lv et al.^33^ reported that the expression of neuropeptide Y (NPY) in the hypothalamic arcuate nucleus (ARC) is regulated by interoceptive signaling through PGE2-EP4 signaling,^34^ thereby inducing adipose tissue lipolysis and promoting osteoblast-mediated bone formation. This mechanism demonstrates that sensory nerves in bone perceive osteoblast-derived PGE2, activating its receptor EP4 as an ascending interoceptive signal to the hypothalamus while downregulating sympathetic tone. Furthermore, elevated PGE2 signaling activates small heterodimer partner-interacting leucine zipper protein (SMILE) and phosphorylated cyclic AMP response element binding protein (pCREB). These two components form a heterodimer that binds to the NPY promoter, suppressing NPY gene expression. This ultimately leads to free fatty acid oxidation and enhanced bone anabolism. Beyond Xu Cao’s PGE2/EP4 work, additional studies confirm skeletal interoception. Gao et al. demonstrated that hypothalamic processing of bone-derived PGE2 regulates weight-bearing bone remodeling and pain in ankle osteoarthritis models.^35^ Lv et al. showed PGE2-EP4 ascending signals downregulate hypothalamic NPY to balance bone-fat metabolism.^33^ Reviews further integrate sensory innervation, COX2-PGE2 pathways, and hypothalamic descending control in bone homeostasis and repair.^36,37^

#### Bone organoids and interoception

Bone interoception refers to the bidirectional signaling along the bone-brain axis between the skeleton and the central nervous system. The bone-brain axis refers to the bidirectional signaling pathway between bone and brain. Bone-derived factors (osteocalcin) can cross the blood-brain barrier and regulate the CNS. On the one hand, these factors support neuronal survival and differentiation, regulate the synthesis of neurotransmitters and therefore influence cognition, memory, and emotional regulation; On the other hand, the function of the central nervous system will in turn regulate skeletal metabolism. The bidirectional cross-system communication indicates bone can not only perform the conventional mechanical roles but can also play crucial endocrine functions in maintaining systemic homeostasis and neural function. Investigating the connection between central nervous system and the bone is essential to understand the complex regulatory mechanisms between bone signaling pathways and interoception. In addition to traditional methods such as in vitro cell cultures and in vivo animal research, the development of bone organoids provides a potentially transformative platform. Bone organoids are in vitro biomimetic models developed through stem cell technology and 3D bioengineering in recent years. The core of their construction lies in the directed differentiation potential of induced pluripotent stem cells (iPSCs) or mesenchymal stem cells (MSCs), by reproducing the natural bone development microenvironment (including BMP, Wnt, and other signaling pathways), these stem cells are guided to self-organize to form 3D structures containing osteoblasts, osteoclasts and mineralized matrix. This kind of model can not only simulate the process of bone formation, mineralization and metabolism, but also be used to study bone related diseases such as osteoporosis, bone dysplasia and mechanisms of fracture healing.^34^ Future research strategies must consider the possibility of using bone organoids or brain organoids to study bone-brain crosstalk; however, functional bone-brain assembloid systems remain underdeveloped.^38^

### Intestinal organoids and interoception

There exists a bidirectional interoceptive communication mechanism between the brain and the gut, known as the gut-brain axis.^39^ This axis involves feedback loops connecting the central nervous system, the gut, and gut microbiota. On the one hand, autonomic regulation of digestion involves the central nervous system, sympathetic nervous system, parasympathetic nervous system, enteric nervous system, and over 20 gut hormones produced by the enteroendocrine system. On the other hand, numerous microbial signaling molecules generated by the gut microbiota (such as lipopolysaccharide) and exogenous/endogenous metabolites derived from food intake (including short-chain fatty acids and bile acids) transmit information to the central nervous system.^40^ The CNS subsequently responds through sympathetic/parasympathetic pathways and the HPA axis. In addition, SCFAs (acetate, propionate, and butyrate) may cross the blood-brain barrier or activate vagal afferents FFAR2/3 receptors, reduce neuroinflammation, promote BDNF expression, modulate serotonin/GABA, and may influence neuroinflammatory processes relevant to neurodegenerative disorders through the gut-brain axis.^34,41^

This gut-brain interaction not only ensures normal gastrointestinal function, but also may influence interoceptive awareness, emotion, and motivation. These effects include not only conscious internal feelings, such as satiety, hunger, pain, nausea, and gastrointestinal discomfort,^42^ but also emotional states such as pleasure and disgust. Evidence shows that gut-brain communication may unconsciously affect mood and motivation, including anxiety and depression.^39,43–46^

Similar to the interaction between the brain and the bones, the intestinal interoception also involves the HPA axis. At present, the research on gut-brain axis mainly focuses on microbiota and microbial metabolites, relying on cell culture in vitro or animal experiments in vivo.^39,43^ Intestinal organoids are cultured in biomimetic extracellular-matrix systems. Intestinal organoids are three-dimensional cultured structures derived from stem cells that mimic the architecture and functions of the human intestine. By culturing ISCs under appropriate conditions, researchers can construct intestine-like organoids in vitro. Unlike traditional 2D cell culture methods, intestinal organoids better reproduce the epithelial architecture and selected functions of the intestine in 3D space, making them a critical tool for studying intestinal biology. These organoids exhibit self-renewal, differentiation, and tissue-specific structural characteristics, enabling them to simulate intestinal epithelial cells, the basal layer, and the microenvironment within the gut. Intestinal organoids reproduce the structure and function of intestinal tissue in vivo through the proliferation and differentiation of ISCs, providing a method for studying the interactions of the intestine at both extracellular and intracellular levels.^47,48^

Intestinal organoids have contributed to drug-screening and toxicology studies. For example, Bao et al.^49^ investigated how carbon nanotubes influence extracellular-matrix mechanics, mitochondrial metabolism, and nutrient absorption in intestinal organoids, illustrating their utility for mechanistic studies of intestinal biology. In a separate context, Wong et al.^50^ used intestinal organoid-based approaches to examine how early-life stress disrupts intestinal homeostasis through NGF-TrkA signaling. Together, these studies support the use of intestinal organoids for analyzing gut homeostasis and stress-responsive pathways, while direct modeling of gut-to-brain interoceptive signaling will require integration with neural, immune, vascular, or microfluidic components.

## Assembloids

Organoids have become valuable models for organ development, disease mechanisms, and drug screening. Individual organoids can contain multiple cell populations, but they generally do not reproduce the long-range neural, endocrine, immune, or vascular interactions required to model communication between organs. Assembloids and multi-organ culture systems may therefore help investigate how stress, intestinal inflammation, skeletal metabolism, and neural function interact across physiological axes.

With the development of organoid research, the concept of the assembloid has emerged. An assembloid is an engineered three-dimensional cellular system generated by fusing distinct organoids or by integrating organoids with additional cell types or tissues through spatial assembly or co-culture. These systems are designed to reproduce complex tissue architecture and intercellular communication and may provide next-generation platforms for studying development, disease, and therapeutic responses. Assembloids can be formed by direct fusion or by microfluidic coupling of organoids in shared or compartmentalized environments while preserving selected tissue-specific characteristics. Potential applications include synaptic circuit formation, neuroendocrine feedback, metabolic signaling, and bidirectional communication between the brain and peripheral organs. Technologies such as calcium imaging, single-cell analysis, and artificial intelligence may further improve the analysis of multi-organ interactions and personalized therapeutic responses (Table 1).

**Table 1:** Types of assembloids

Assembloids	Method	Target	Relevance to interoception
Multi-region assembloids	iPSC-generated multi-region brain organoids	Combine distinct brain-region organoids	Paracrine signaling VEGF, BMP, PDGF, IGF signaling, etc.
Multi-organ assembloids	iPSC-derived heart/liver/kidney	Vascularized co-culture	Systemic homeostasis, drug metabolism, interoceptive axes
Neural assembloids	Corticostriatal-spinal-muscle assembloid	Model components of ascending sensory pathways	Neuro-somatosensory pathways
Bone assembloids	MSCs/hPDCs microtissue-hydrogel hybrids	Model skeletal maturation and innervation	Bone-ECM signaling with interoception

### Assembloids with interoception

#### The development of assembloids

Organoids aim to improve traditional two-dimensional culture systems and animal models that simulate physiological functions of human organs. Organoids are derived from the three-dimensional self-organizing capacity of pluripotent stem cells or adult stem cells, which can more accurately simulate organ development and disease progression. However, traditional organoids lack complex interactions between regions or organs, which are crucial for simulating physiological processes, including interoception.

The concept of three-dimensional organization self-assembly can be traced back to 1907, when Wilson demonstrated that individual cells (isolated cells) of sponges could reorganize to form functional structures. Later, Weiss et al. successfully constructed tissue-specific structures using single-cell suspensions of embryonic organs, laying the foundation for contemporary organoid research. The discovery of embryonic stem cells in the 1980s and the emergence of induced pluripotent stem cells (iPSCs) by Takahashi and Yamanaka in 2006 were milestones in organoid research. In 2009, Sato et al. cultured intestinal organoids from Lgr5^+^ stem cells, providing a solid foundation for self-renewal and morphogenesis of epithelial cells.

With the deepening of organoid research, its limitations are gradually becoming apparent. Because most organoids represent a restricted tissue context and cannot simulate multicellular interactions or systemic signaling, researchers have begun to integrate multiple organoids, giving rise to the concept of “assembloids”. The term “assembloid” was introduced by Paşca et al.,^51^ who defined this type of composite three-dimensional culture as a structure formed by the fusion of organoids representing multiple regions, or by the addition of other types of cells (such as glial cells, immune cells, or endothelial cells) to multiple regions of organoids.

Multi-region assembloids have enabled investigation of neural processes such as subdomain-specific forebrain spheroids,^52^ thalamus-like brain organoids^53^, and cortico-motor assembloids,^54^ showing how fusion of physically separated organoids can recapitulate long-range cell migration and axon pathfinding for brain development and interoception. Multi-lineage assembloids can address limitations of organoid culture, such as a lack of key supporting cells. For instance, co-culturing brain organoids with microglia or endothelial cells allowed modeling of immune-neural interactions or neurovascular barrier formation. Technological innovations in microfluidics, spatial bioprinting, or synthetic scaffolds may improve the fidelity of organoid assembloids and long-term co-culture. Recent studies have also proposed assembloid strategies for bone and cartilage. In the case of skeletal model systems, for example, constructed assembloids that co-cultured mesenchymal stem cells, chondrocytes, and vascular-like networks have been applied to examine tissue maturation, mechanical and inflammatory feedback, essential to bone interoception.^38^ Such models may be incorporated into neural components to model nociceptive or proprioceptive neural circuits between skeletal and neural circuits. Beyond brain-bone-gut, assembloids have been generated for heart-liver (vascularized self-assembly), lung-airway, and kidney models to study inter-organ signaling.^55^

In conclusion, assembloid development represents a paradigm shift in tissue engineering. Assembloids are the next step after organoid models, providing a flexible platform for the emulation of inter-organ communication and the complexity of physiological phenomena. Signal transduction, regional specialization, and multicellular interactions are some examples of useful aspects to study when considering these interactions.

### Research on brain-related interoception using assembloids

**Fig. 4:**
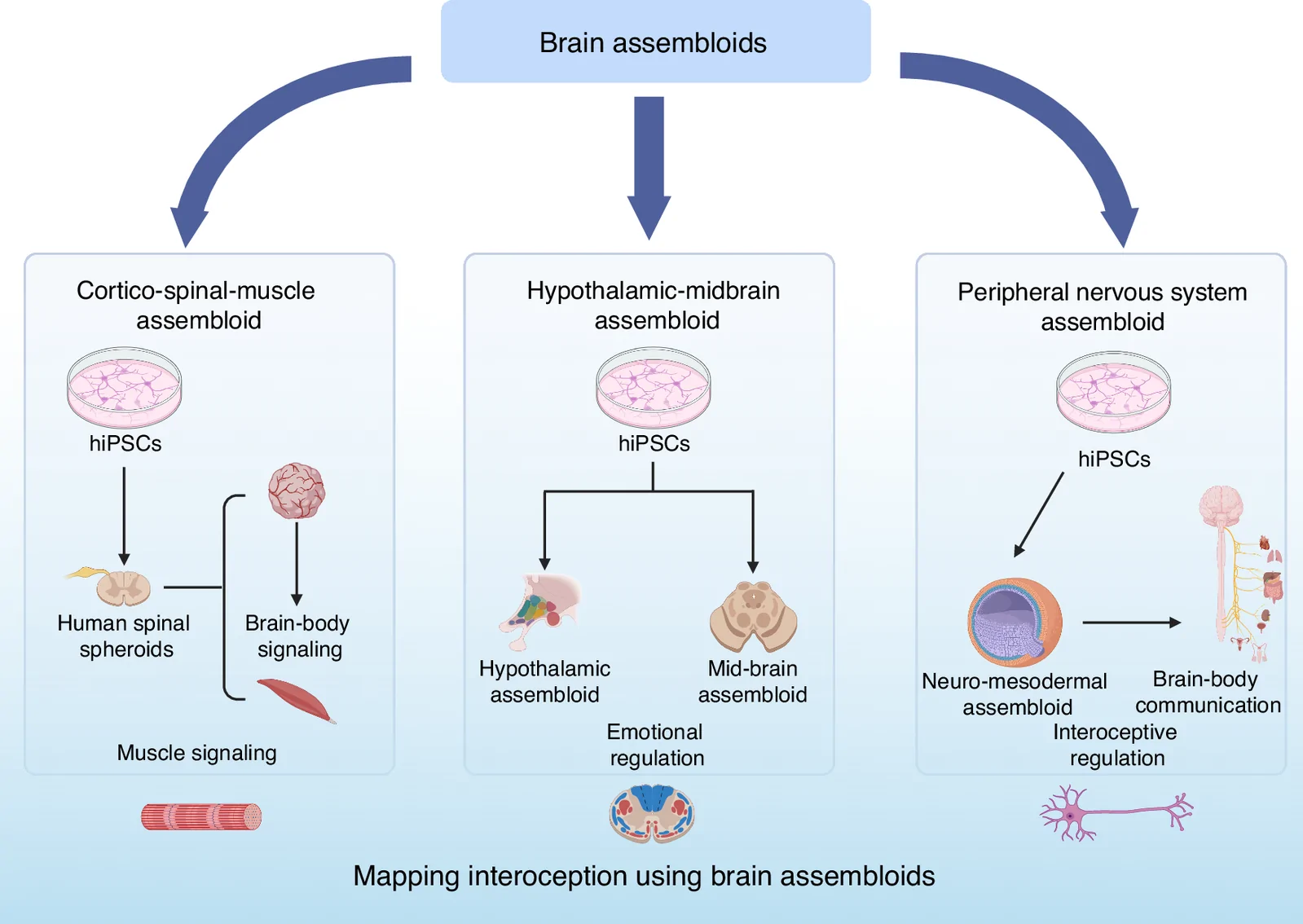
Brain assembloid platforms relevant to interoception research. Reported cortico-motor and thalamocortical assembloids provide models for neural circuit assembly and sensorimotor integration, whereas cortico-hypothalamic and peripheral organ-brain systems remain prospective platforms for studying neuroendocrine and interoceptive regulation

Interoception refers to the perception of signals from within the body. These signals originate from organs, hormones, immune cells, and other internal systems, which are perceived and help the brain control bodily functions such as stress, hunger, pain, and body temperature. In recent years, brain organoids have provided us with an effective method for understanding brain development, but traditional brain organoid models have limitations in studying the connection between the brain and the body. Researchers have developed assembloid approaches, which constructs brain assembloids by fusing various types of organoids to simulate brain circuits and brain-body interactions. As a novel brain model, brain assembloids can provide better tools for studying how the brain receives and responds to internal signals (Fig. 4).

An example is the cortico-motor assembloid. This model connects cortical organoids with spinal cord organoids and muscle tissues. It creates a pathway from the brain to the body, demonstrating how the brain issues motor commands and how the body transmits feedback information to the brain, which is related to interoception. The cortical-motor assembloid proposed by Andersen et al.^54^ is a three-dimensional composite assembloid system constructed from human induced pluripotent stem cells (hiPSCs), aimed at simulating neural pathways from the cerebral cortex to control skeletal muscle movement.^56^ The key lies in reproducing the multisynaptic neural circuits from the cerebral cortex, spinal cord to the terminal effector muscles, thereby achieving autonomous musculoskeletal control. It contains three region-specific organoids derived from hiPSC: the human cortical sphere (hCS) representing the cerebral cortex, the human spinal cord sphere simulating the human posterior and cervical spinal cord, and the human skeletal muscle sphere representing mature human skeletal muscle. These components are spatially assembled in a cortico-spinal-muscle sequence, establishing synaptic connections that form a functional cortico-motor circuit. In future research, additional sensory neurons and dorsal root ganglion cell populations can be utilized to simulate pain, stress, and motor related signals transmitted from the body to the brain—a model that is highly useful for studying diseases such as amyotrophic lateral sclerosis (ALS) and testing new therapies.

Another important model is the thalamocortical assembloid.^57^ It connects a thalamic organoid with a cortical organoid. The thalamus receives sensory information from the body and sends it to the brain. The cortex and hypothalamus play key roles in regulating complex physiological functions such as feeding behavior, sleep-wake cycles, thermoregulation, emotional responses, and neuroendocrine axis activity. However, this is difficult to replicate accurately in animal models. A possible solution is to form a combination of cortical and hypothalamic organoids through fusion.

Region-specific organoids derived from human pluripotent stem cells, including iPSCs, can be assembled under three-dimensional co-culture conditions. In a prospective cortico-hypothalamic model, cortical projection neurons could extend axons towards hypothalamic neurons and form long-range synaptic connections. Such a system would provide a potential platform for investigating neuroendocrine regulation; however, its functional validation for interoceptive signaling remains to be established.

Although a fully mature cortico-hypothalamic assembloid model has not yet been reported, such systems can provide new tools for studying diseases involving impaired cortico-hypothalamic connectivity, including autism spectrum disorders, eating disorders, and affective disorders. In addition, this model has the potential to study how the cerebral cortex regulates the hypothalamic behavioral center, such as interoception, which refers to neural sensing and integration of internal bodily signals.

In summary, the core mechanisms of the cortico-hypothalamic assembloid include region-specific self-organization, spontaneous long-range axonal projection, synaptic integration, and potential integration of neural and neuroendocrine functions. Although the model is still in the conceptual stage, it provides a new platform for studying the physiological mechanisms between brain-body communication and emotions, metabolism, and in vivo states. It can explore how internal signals such as inflammation or intestinal discomfort affect brain activity and are also applicable for studying diseases such as epilepsy or autism.

In addition, cortico-striatal-midbrain assembloids were constructed. These systems connect cortical organoids with midbrain organoids containing dopaminergic neurons. The midbrain is responsible for regulating rewards, motivation, and energy expenditure. The assembloids developed by Levy et al.^58^ and other researchers simulated this neural circuit. These models can demonstrate how the brain responds to signals such as hunger or stress, which is of great value for studying Parkinson’s disease, depression, and addiction disorders, which often accompany abnormal responses of the brain to internal states. Assembloids help to understand how inner feelings affect emotions and decision-making processes.

Some researchers are combining brain organoids with peripheral organoids such as intestines and bones. These systems can study the direct communication mechanism between the brain and the body. For example, researchers use brain-gut assembloids to observe signals transmitted from the gut to the brain,^59^ including microbe-gut-brain signaling.^19^ These models help explain stress-related diseases and brain-immune interactions. Currently, there are still many challenges to be faced. Due to the varying growth rates of different types of organs and the fact that these systems often lack blood vessels or immune cells, it is difficult to form stable connections. A major technical hurdle in assembloids is media compatibility: brain cells require neurotrophic factors (BDNF, NT-3) and low serum, bone cells need high calcium/phosphate and BMPs, while intestinal cells demand EGF/Wnt/R-spondin gradients. Mismatched media causes cell death, dedifferentiation, or biased growth. Microfluidic gradient devices and AI-optimized formulations are emerging solutions.^60^

Currently, new tools such as microfluidic chips are assisting in model optimization. In the future, brain organoid-based systems will be more suitable for interoception research, helping to explain how the brain perceives body states and maintains internal system balance.

#### Research on bone using assembloids

Bones are not only structural tissues, but also play a positive role in hormone release, immune function, and communication with the nervous system. In recent years, researchers have successfully constructed three-dimensional bone organoids that simulate the partial structure of native bone tissue. These models can generate bone matrix and support bone cell development, but they cannot fully present the interaction mechanism between bones and other organs. To address this issue, scientists have begun constructing bone assembloids that combine bone organoids with other types of organs, such as brain organoids. This new type of model helps us understand how the brain receives and responds to internal signals from bones.^6,31^ There are studies indicating that bone assembloids may have practical value in studying the skeletal interoception. Xu Cao et al. proposed that PGE2 may affect brain regions such as the hypothalamus.^34^ These signals may change during bone injury, mechanical loading, or aging processes, and may affect the brain’s regulation of stress, metabolism, or immune responses. The brain-bone assembloids enable researchers to understand this pathway in vitro, providing a controllable experimental platform for observing signal transduction and its impact on brain activity. This model also helps to understand the disease mechanisms of bone-brain interaction, such as osteoporosis and chronic inflammation.^29^

Bone organoids are prepared from stem cells such as iPSCs or periosteum-derived skeletal stem/progenitor cells. Bone organoids can form small, mineralized areas, develop early bone cells, and even contain vascular-like supporting structures.^33,34^ When fused with brain or nerve cells, this system can be used to study the brain’s response to changes in the skeletal environment. For example, integrating autologous nerve grafts into biomaterials to form implant materials that promote the formation of nerve tissue and bone tissue. In the future, such assembloids can further expand immune cell or vascular structures, thereby constructing more complete models of the bone microenvironment and its interaction mechanisms with the brain.^33,34^

There are still several challenges at present. Bone and brain tissue have different growth rates and varying nutritional requirements, making it difficult to maintain tissue connectivity between the two in the same culture system. In addition, most existing methods lack stability. Microfluidic systems, bioengineering scaffolds, and 3D bioprinting are expected to solve these challenges.^61^ By improving these tools, bone assembloids may become a key method for studying how the brain perceives signals inside bones and how the body maintains balance through brain-bone communication.

#### Research on intestinal using assembloids

The gut is an important organ in interoception because it continuously transmits information to the brain about digestion, nutrient absorption, and immune response. Traditional intestinal organoids are mainly composed of epithelial cells, which cannot simulate the complex interaction between organs. To address this limitation, researchers have developed gut assembloids that combine gut organoids with other cell types. These methods help to reconstruct the gut environment more effectively, making it possible to study how the gut interacts with other organs. Micati et al.^21^ found that co-culture of intestinal organoids with stromal cells can promote the growth and maintenance of epithelial cells and help reproduce the villous structure of intestinal crypt-villus architecture. Mesenchymal cells provide signals, including WNT, BMP antagonists, and EGF, to maintain stem cell characteristics and promote epithelial regeneration after injury. Some organoid models contain a mixture of intestinal organoids and mesenchymal cells or specific extracellular matrix, forming self-organizing structures with compartments of stem cells and differentiated cells. This bidirectional communication is crucial for studying how the gut perceives and responds to injury or inflammation and is an important component of interoception.^21^

In addition, some studies have applied intestinal organoids to simulate intestinal inflammation and immune defense. For example, pro-inflammatory cytokines TGF-β1 or PGE2 can be added to organoid cultures to simulate the intestinal inflammatory response that occurs in inflammatory bowel disease (IBD), thereby helping to gain a deeper understanding of the gut-brain sensory pathway.^62^ Chen et al.^63^ emphasized the necessity of constructing multi-organ organoid systems to achieve inter-organ communication. They believe that true intestinal function involves not only epithelial cells, but also connections with the nervous system, immune system, and vascular system. For example, by co-culturing gut organoids with neural modules derived from neurons or iPSCs, we can reconstruct gut−brain communication circuits whose functions are crucial for the etiology of diseases such as irritable bowel syndrome or gut-driven emotional disorders.

Organ-on-a-chip systems have also been used to more accurately simulate the gut environment. In recent studies, human intestinal organoids were placed on microfluidic chips to simulate blood flow and nutrient gradients. These chips allow co-culture with vascular endothelial cells and immune cells. For example, Mitrofanova et al. developed a miniature colon model using a collagen matrix gel scaffold and a controlled growth factor gradient, which successfully reconstructed the stem cell microenvironment at the bottom of the artificial crypt while promoting the differentiation of apical cells into resorptive and goblet cells. Such systems enable investigation of how gut signals are processed and how the gut interacts with immune and nervous systems during stress, infection, or nutrient sensing.

Colorectal organoids have also been used to model intestinal disease. Patient-derived epithelial organoids described by Dotti et al.^62^ retained disease-associated epithelial alterations in ulcerative colitis, supporting their use for studying mucosal pathology. This study did not establish a colon assembloid model of colorectal cancer; modeling immune-brain communication or stress signaling would require further integration with immune, neural, vascular, or microfluidic components.

Intestinal organoids represent an important advance in the field of interoception. By combining gut organoids with matrix, immune, neural, and vascular components, researchers can explore gut-brain communication mechanisms, which are involved in homeostatic regulation, inflammation, and disease.

## Interoception-related diseases

### Interoception in depression, anxiety, and autism spectrum disorder

**Fig. 5:**
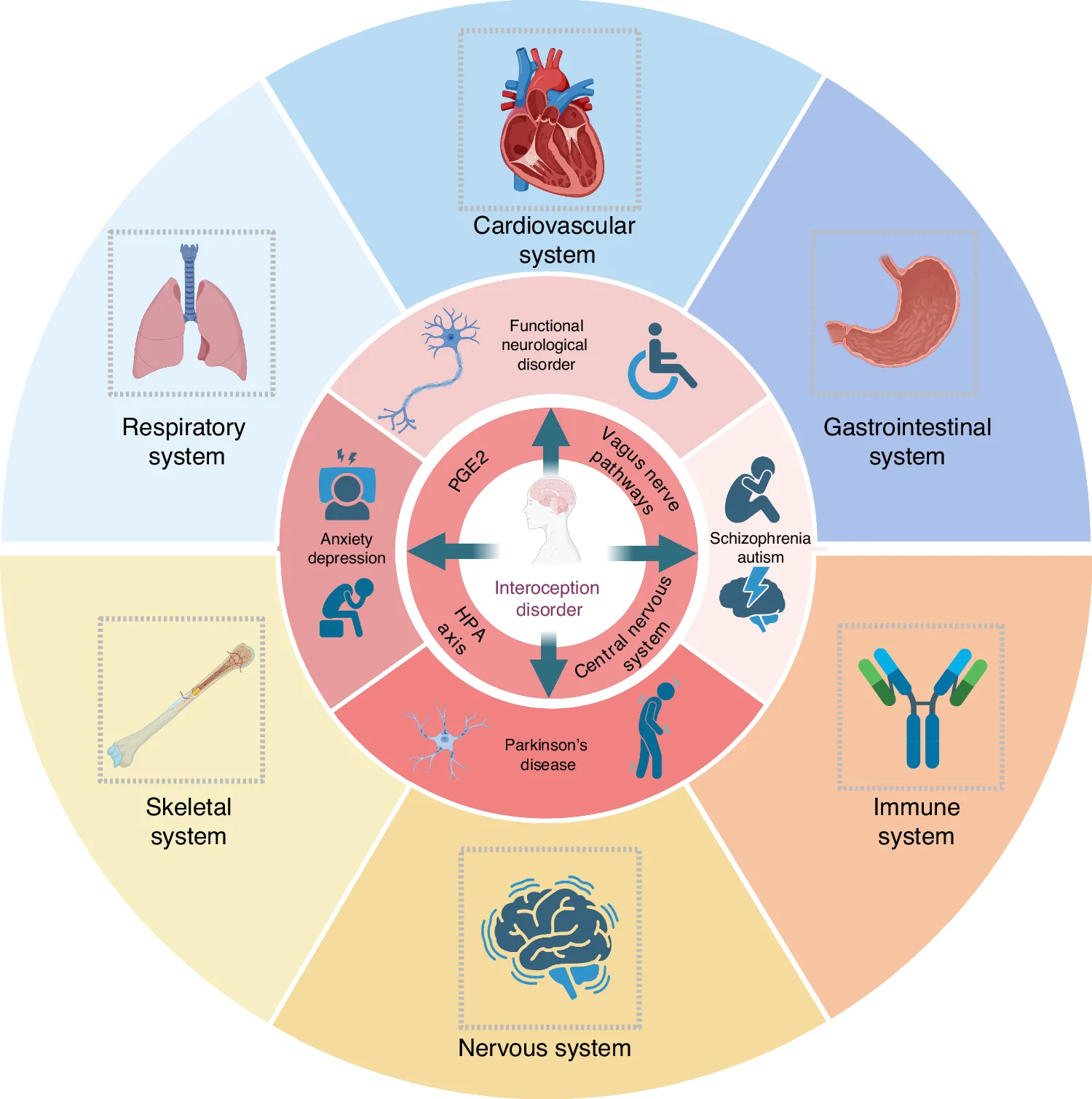
Interoceptive dysfunction across multisystem disorders. Altered signaling along gut-brain, skeletal-brain, cardiovascular, respiratory, immune, and neural pathways may contribute to abnormal interoceptive processing in neurological and psychiatric disorders. Arrows indicate candidate signaling routes rather than established causal relationships in all listed diseases

Interoception is the ability of the brain to perceive and interpret signals from within the body. These signals come from multiple sources, such as the cardiovascular system, respiratory system, immune system, gastrointestinal tract, and musculoskeletal tissues. Recent studies have shown that interoceptive dysfunction is associated with a variety of neurological and psychiatric disorders. This connection provides a new perspective for understanding and treating diseases such as functional neurological disorders (FND), anxiety, depression, schizophrenia, autism, Alzheimer’s disease, and Parkinson’s disease^42,64–66^ (Fig. 5).

FND is a condition in which patients have symptoms such as tremor, weakness, seizures, or speech disorders, that are not fully explained by structural neurological disease. Jungilligens et al.^67,68^ proposed that changes in interoceptive processing and emotional construction play a key role in FND. The brain predicts and interprets body signals to construct emotional categories. When this process goes wrong, it can lead to physical symptoms that are difficult to explain with standard neural models. For example, people with FND may misinterpret internal signals associated with arousal, stress, or energy regulation, leading to dizziness, numbness, even paralysis. These symptoms are not imaginary but rather misrepresent the state of the body due to real changes in interoception processing.

Dysfunction in sensory processing is commonly seen in psychiatric disorders such as post-traumatic stress disorder, depression, and anxiety. Nord and Garfinkel’s research^69,70^ suggests that patients may excessively focus on or misinterpret physiological signals, leading to sustained negative emotional states. For example, patients with anxiety disorders are often highly sensitive to heartbeat and breathing. This phenomenon can form a vicious cycle, causing them to feel fear and uncertainty about their physical sensations, which further exacerbates the symptoms. Patients with depression often experience internal sensory abnormalities, manifested as reduced sensitivity to internal signals such as hunger and fatigue, leading to emotional numbness and decreased motivation. These findings suggest that interventions targeting interoceptive pathways may be an effective treatment strategy that can be combined with medication, breathing training, mindfulness therapy, and other behavioral therapies.^16^

Neurodegenerative diseases and neurological disorders are also clearly associated with sensory dysfunction. Bonaz et al.^71^ proposed that Parkinson’s disease (PD) is considered a systemic disease driven by gut-brain axis disorder. Gut microbiome imbalance may increase intestinal mucosal permeability, and local inflammation can induce early abnormal accumulation of α-synuclein in the enteric nervous system, which may spread retrogradely to the central nervous system via the vagus nerve, thereby promoting neurodegenerative changes. Alzheimer’s disease patients often experience impaired cardiac sensations, making it difficult to monitor their own physiological status, which may be related to the common loss of self-awareness and emotional regulation disorders in dementia. Because of damage to the insular cortex and anterior cingulate cortex, autism spectrum disorder (ASD) also suffer from interoceptive dysfunction: The patient’s perception of internal signals such as heartbeat is less accurate, but the subjective perception of physical sensation is enhanced. This imbalance between actual feelings and perceptions may lead to anxiety, social difficulties, and sensory overload, making it difficult for patients to identify and describe emotions, thus further exacerbating the difficulties of emotional communication and social interaction. These interoceptive impairments may underline features of autism, such as impaired emotional expression.^72^

In the field of treatment, enhancing interoceptive processing has become another research direction. Mindfulness training, breathing exercises, and vagus nerve stimulation have been used to restore physical and mental balance. Slow breathing can activate the parasympathetic nervous system response, thereby reducing negative emotions and promote emotional regulation. In addition, drugs such as selective serotonin reuptake inhibitors (SSRIs) may indirectly affect interoceptive processing by modulating activity within interoceptive networks, including the insular cortex. Elucidating the role of interoception in neurological disorders may help design personalized treatment plans, stratify based on specific interoceptive characteristics of patients, and determine the optimal treatment approach.

Interoception is not only the background process to maintain homeostasis, but also the core mechanism for the brain to construct emotional and physical experience. Disturbance of interoception can lead to a variety of neurological and psychiatric disorders. Integrating interoception into neurological disease models will provide a new approach for the diagnosis, treatment, and prevention of brain-body dysfunction.

### Interoception in gut disorders

Interoception plays a key role in regulating gastrointestinal function and maintaining communication between the gut and the brain. Signals from the gastrointestinal tract, such as mechanical tone, nutrient composition, pH, and microbial metabolites, are transmitted to the brain via vagal and spinal afferents. These signals are processed in the nucleus of the solitary tract, the parabrachial nucleus, the insular cortex, the anterior cingulate cortex, and the hypothalamus, forming a core neural circuit for interoception.^14^

Gut-brain interaction disorders are strongly associated with interoceptive dysregulation, including irritable bowel syndrome (IBS) and functional dyspepsia. Patients often show visceral hypersensitivity, increased pain perception, and emotional dysregulation. Neuroimaging studies have shown increased activation of the insular and anterior cingulate cortex upon visceral stimulation in IBS, indicating abnormalities in the central processing of gut signals. Stress, early life trauma, and psychosocial factors may further exacerbate sensory changes through HPA axis dysfunction.^21,57^

The vagus nerve is the main pathway for sensory signals from the gut to the brain. Studies have found that patients with irritable bowel syndrome, anxiety disorders, and depression all show impaired vagal tone, suggesting a common pathway between gastrointestinal and mood symptoms.^12,14,17,19^ This common mechanism is reflected in the high comorbidity of intestinal diseases and psychiatric diseases, which may result from the abnormality of interoceptive prediction and regulation.

The gut microbiota is another key factor affecting intestinal sensory function. Microbial metabolites, such as short-chain fatty acids, and neuroactive molecules or precursors, including GABA and 5-HT, can regulate gut and central nervous system activity. Microbial dysregulation is associated with altered vagal signaling, enhanced inflammatory responses, and symptoms of gastrointestinal disease and mood disorders.^12,14,17,19^ These results suggest that microflora is a key regulator affecting the accuracy of gut-brain signal transmission and signal transduction.

Patients with functional gastrointestinal disorders (FGIDs) often have increased intestinal permeability and mild mucosal inflammation.^66^ These immune alterations can activate sensory afferents and sensitize the interoceptive system, resulting in heightened perception of intestinal stimuli.^19^ Changes in interoception triggered by inflammation may also affect the gut-brain interoceptive circuit and thereby influence mood and behavior.

Interventions that target interoceptive processing have emerged as promising treatment. Mindfulness training and vagal stimulation have been shown to improve symptom severity and visceral awareness in irritable bowel syndrome and related disorders.^4,7^ Drugs such as semaglutide and tirzepatide can slow gastric emptying and suppress appetite and may modulate the transmission of interoceptive signals but may also cause nausea, vomiting, or constipation, suggesting the need for more selective interoceptive modulation.^46^

Despite these advances, tools to assess gut-specific interoceptive dysfunction are currently lacking, and there is still controversy regarding the causal relationship between interoceptive dysfunction and gastrointestinal pathology. Future studies on the integration of organoids may provide new insights into the pathophysiology of intestinal diseases.^19,21^

### Skeletal interoception

Interoception not only regulates visceral function but also plays a key role in regulating skeletal homeostasis. Emerging evidence indicates that bones are not only structural organs, but also dynamic sensors that form a closed-loop communication system with the central nervous system through neural circuits.^31,33^ Skeletal sensory pathways enable bones to transmit physiological status information to the brain, thereby regulating physiological activities including bone metabolism, inflammatory response, and bone regeneration.

One mechanism of skeletal interoception involves the release of PGE2 by osteoblasts in response to mechanical loading or bone injury. This molecule binds to EP4 receptors on sensory nerve endings in the bone marrow, initiating interoceptive signals to the hypothalamus. The ventromedial hypothalamus (VMH) is activated via cAMP/CREB signaling pathway, resulting in reduced sympathetic output. Reduced sympathetic tone may promote bone remodeling and repair by enhancing osteoblastic differentiation of MSCs and supporting bone formation.

In addition, this feedback is not only involved in homeostatic regulation but also plays a key role in bone disease. For example, impaired PGE2 signaling or sensory neurodegeneration may cause delayed fracture healing and osteopenia. It may involve the impairment of interoceptive circuits. These disorders have been observed in aging and chronic diseases such as osteoporosis, arthritis, and degenerative spinal diseases.^31,33,34^ In addition, inflammatory cytokines may further enhance the sensitivity of bone pain receptors, and contribute to chronic skeletal pain such as osteoarthritis.^29,64^

Recent studies reveal sclerostin, an osteocyte-derived Wnt inhibitor, participates in the bone-brain axis and promotes Alzheimer’s disease (AD). Bone-derived sclerostin crosses the blood-brain barrier, dysregulates Wnt/β-catenin signaling, increases amyloid-β (Aβ) production, impairs synaptic plasticity, and accelerates cognitive decline in aged and AD mice.^73^ Serum sclerostin levels correlate positively with brain Aβ burden and negatively with cognition in elderly and AD patients.^74^ Anti-sclerostin therapy ameliorates both bone loss and memory impairment, positioning sclerostin as a therapeutic target in bone-brain interoceptive disorders.

Interoceptive pathways provide potential therapeutic targets, activating the PGE2-EP4 sensory axis or regulating the hypothalamic responses may enhance bone regeneration capacity. For example, EP4 agonists have been shown to accelerate bone repair in animal models.^34^ These findings suggest that utilization of interoception pathways provides a new strategy for the treatment of bone diseases.

In addition, organoids and assembloids are being developed to simulate in vitro bone-nerve-brain interactions, providing a platform for mechanism research and drug screening.^31,33,34^ These models may ultimately enable the design of precise treatment plans, restoring bone health by rebalancing sensory circuits throughout the body.

## Organoids and interoception: future prospects

Organoids provide a superior culture paradigm for organogenesis, signaling pathway characteristics, and pathological studies of various diseases in different organs relative to conventional two-dimensional cell culture models, which is helpful for the development of new drugs and exploration of disease mechanisms. This review highlights the potential new directions in organoid research, with a focus on exploring the potential connection between organoids and interoception (i.e., bidirectional communication between the internal organs and the central nervous system). However, there is direct functional evidence linking organoid systems to interoceptive circuits remains limited. Therefore, further research is needed to clarify their relationship. In addition, brain organoids generally remain developmentally immature, often resembling prenatal stages, and it remains challenging to establish a complete functional connection between target organs and interoception. In the future, more mature brain organoids will need to overcome these limitations. In summary, establishing the connection between visceral organs and the sensory system within the central nervous system will help open up new avenues for pathological research on diseases related to the central nervous system, bones, and gastrointestinal system.

With the development of our understanding of interoception and the brain’s ability to perceive and regulate the state of the body, the development of experimental platforms that can model, measure, and perturb visceral signals and integrate them with brain activity is attracting increasing attention. The development of organoid technology has opened up a new way to study the dynamics of interoceptive pathways. In the field of brain science research, brain organoids have been demonstrated to reproduce the early features of neurogenesis, forebrain regional patterning, and cortical organization. Recent studies using microcolumn array chips to standardize organoid production and achieve dynamic stimulation through exogenous signals such as cytokines or tumor-derived exosomes provide promising research methods for understanding how peripheral inputs affect central interoceptive processing.^7,52,75^

Microelectrode arrays (MEAs) and biosensors can expand the analytical capabilities of these systems. MEAs can record the characteristics of neural activity in brain organoids in real time as a quantitative readout of interoceptive signal output. Additionally, biosensors can detect physiological status related to functions, such as pH, neurotransmitter, or inflammatory cytokines. These sensors not only enable precise monitoring of the physiological status of organoids but also serve as critical input channels in closed-loop neuromodulatory systems.^43,50,76^ Strategies to enhance organoid maturity include long-term culture to reach postnatal-like stages and chronic electrical pulse stimulation (EPS) via microelectrode arrays, which activates CAMKII-PKA-pCREB pathways, promotes cortical layering, synaptic density, and electrophysiological maturation in brain and neuromuscular organoids.^77,78^

AI has been deeply integrated into the medical field and widely involved in biological signal feature extraction and functional change prediction. These technologies have been applied to disease diagnosis, management and prediction. For example, in the case of spinal cord injury or other neurological diseases that lead to the failure of interoception pathways and urinary dysfunction, intelligent neural units and machine learning algorithms have been developed that can monitor bladder pressure and filling status through the afferent nerve activity of conventional bladder nerve roots.^79^

In the field of organoid research, scientists have proposed the innovative concept of organoid intelligence (OI). This innovative technology utilizes microfluidic perfusion systems and brain organoids containing glial cells for biocomputing. This forward-looking idea aims to connect brain organoids with various sensors and input-output devices to build complex communication interfaces. By combining biofeedback, big data and artificial intelligence training technology, the goal is to establish a new generation of standards for biocomputing systems to deal with diseases such as cognitive impairment.^80^

AI can also identify dynamic physiological patterns and pathological signatures. These functions can, in turn, enable automated feedback mechanisms, such as electrical stimulation, targeted drug delivery to modulate organoid behavior in real time. AI-assisted organoid-on-chip systems have the potential to predict interoception-related pathways across systems and could serve as high-throughput platforms for personalized medicine and drug discovery.^59,60,80^ Machine learning models (e.g., Random Forest for feature importance and Neural Networks for time-series analysis) can be trained on multimodal data (electrophysiology, metabolomics, and pH) to decode interoceptive prediction errors, defined as discrepancies between expected and observed signals in shared environments. For instance, media incompatibility (differing nutrient/growth factor demands) manifests as anomalous calcium spikes or metabolic shifts; anomaly detection algorithms flag these as interoceptive errors, enabling real-time feedback via microfluidic adjustments, directly linking biological predictive coding to computational optimization in organoid intelligence (OI) systems.^80,81^

**Fig. 6:**
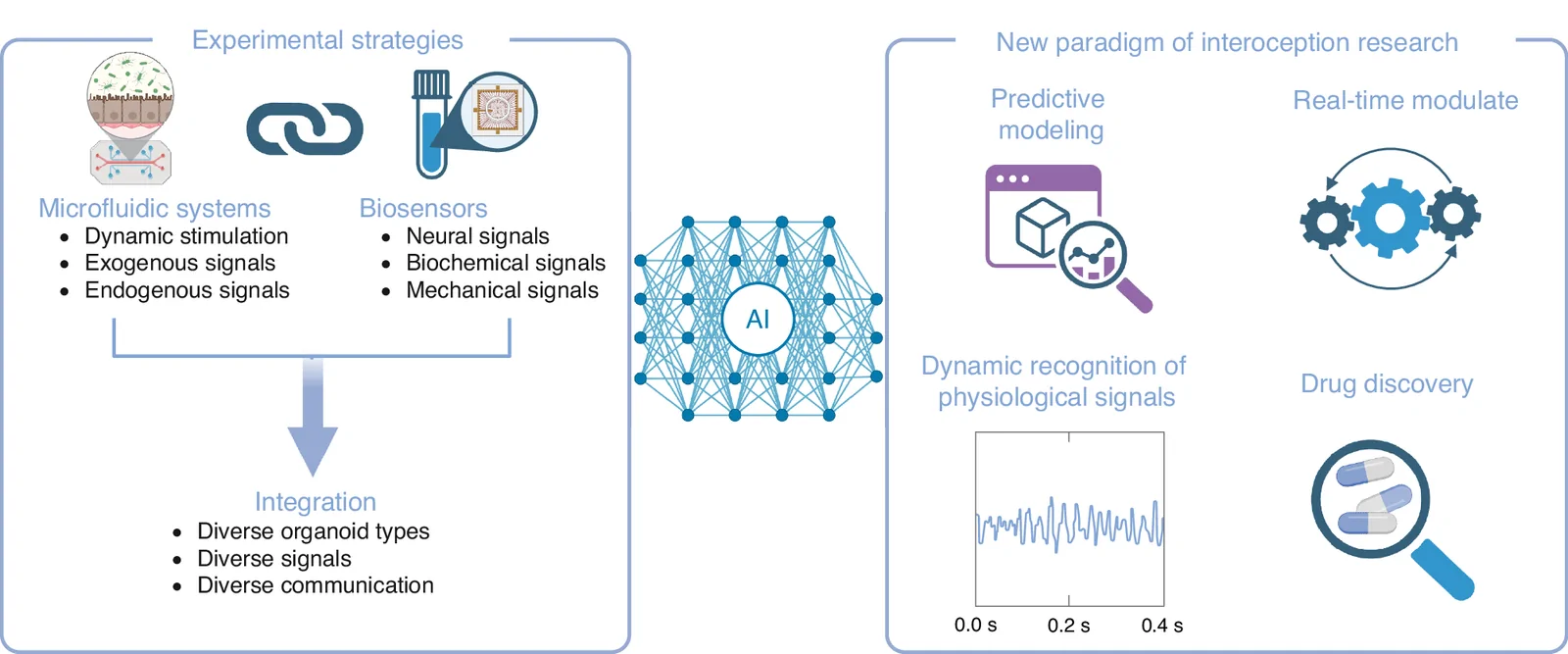
Future directions for organoid-based interoception research. Organoid-on-chip platforms, biosensors, and AI-assisted analysis may be integrated to measure biochemical, mechanical, and electrophysiological signals and to guide hypothesis-driven perturbation experiments. These approaches could support mechanistic studies of brain-body communication, drug discovery, and personalized modeling, provided that biological validation and uncertainty assessment are incorporated

Future work should integrate multiple types of organoids or assembloids to simulate organ communication, as well as using machine learning or deep learning algorithms to achieve gene-level prediction. These solutions lay the foundation for constructing intelligent organoid platforms, which can analyze the molecular mechanisms of interoception and achieve the integration of neuroscience and AI through intelligent methods (Fig. 6).
